# Middle-Preserving Pancreatectomy for Multicentric Solid Pseudopapillary Neoplasm in a 10-Year-Old Female

**DOI:** 10.1055/s-0044-1791812

**Published:** 2024-10-17

**Authors:** Grace Marshall, Matthew Byrne, Korry Wirth, Xiaoyan Liao, David C. Linehan, Nicole A. Wilson

**Affiliations:** 1 University of Rochester School of Medicine and Dentistry, Rochester, New York; 2 Department of Surgery, University of Rochester Medical Center, Rochester, New York; 3 Department of Pathology and Laboratory Medicine, University of Rochester Medical Center, Rochester, New York; 4 Department of Surgery, University of Rochester Medical Center, Rochester, New York; 5 Departments of Surgery, Pediatrics, and Biomedical Engineering, University of Rochester Medical Center, Rochester, New York

**Keywords:** multicentric solid pseudopapillary neoplasm, Frantz tumor, urogenital abnormalities, pediatric, pancreas

## Abstract

Solid pseudopapillary neoplasm (SPN) is a rare low-grade malignant tumor of the pancreas that occurs predominantly in young females. This tumor is occasionally multicentric, posing a unique surgical conundrum for resection. We present a case of a 10-year-old female with a history of multicystic dysplastic left kidney and persistent urogenital sinus who was diagnosed with biopsy-proven multicentric SPN of the pancreatic head and tail and underwent middle-preserving pancreatectomy. The patient tolerated the surgery very well. Our case is one of the few reported cases of multicentric SPN in a pediatric patient, and the only case treated with middle-preserving pancreatectomy, which is a novel surgical option for protecting pediatric patients from total endocrine and exocrine pancreatic insufficiency. With the increase in the incidence of SPN, there is an increasing need for pancreas-preserving surgical options, particularly in pediatric patients.

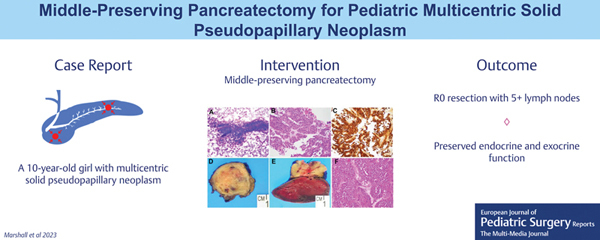

## Introduction

Solid pseudopapillary neoplasm (SPN) is a low-grade malignant tumor of the pancreas with excellent clinical course. It was first described by Frantz in 1959 and was originally called a “Frantz tumor.” Although the histogenesis is still uncertain, there are some hypotheses regarding the tumor cell origin. 1 2 SPN represents 0.9 to 2.7% of pancreatic neoplasms and 5% of cystic pancreatic neoplasms. It typically occurs in young females, with a female-to-male ratio of 9.8:1 and a mean age of 28.5 years at presentation in the general population. 1 In pediatric patients, SPNs are the most commonly encountered pancreatic tumor, usually found incidentally 1 3 at a mean age of 13.6 years. 4

SPN is typically a solitary lesion located in the body/tail (54%) or head of the pancreas (46%). 4 Multicentricity is rare, with fewer than 15 cases described in the literature and only a single previously reported multicentric case in a pediatric patient. Unlike pancreatic duct adenocarcinoma, patients rarely present with symptoms of biliary or pancreatic obstruction. 4 Common symptoms include abdominal pain, dyspepsia, and early satiety. 3 Laboratory workup typically shows normal tumor markers.

Management is through surgical resection, and patients typically have excellent prognoses with long-term survival. 3 4 5 6 Few factors predict recurrence or metastasis, including the presence of a pancreatic head tumor, which has been linked to worse outcomes. 7 Of the 15 previously reported cases of multicentric SPN, the single pediatric patient was treated with enucleation in the head and tail. 8 Middle-preserving pancreatectomy (MPP) has been described as a viable surgical option for patients with pancreatic head and tail tumors. 9 There are at least two case reports of MPP in the literature for SPN, both in adults. 9 Herein, we present a unique case of multicentric SPN in a pediatric patient treated with MPP consisting of distal pancreatectomy, splenectomy, and classic pancreaticoduodenectomy.

## Case Report

**Fig. 1:**
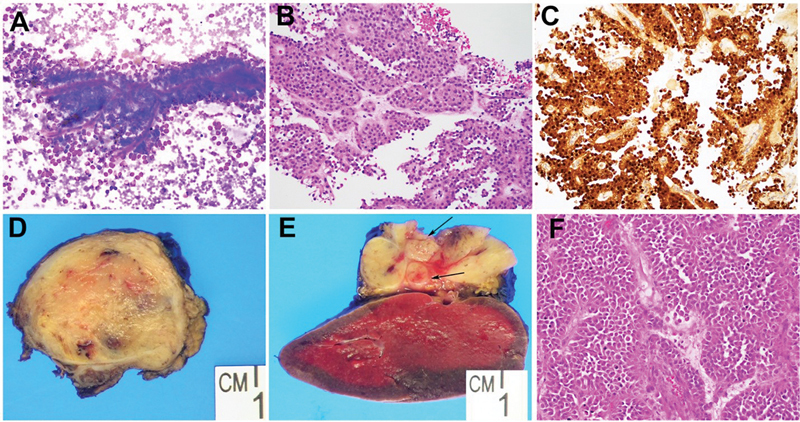
Cytology and histology of this rare multicentric solid pseudopapillary neoplasm (SPN). ( **A** ) Fine needle aspiration of the pancreatic head mass showing cellular smears with delicate papillary fronds. ( **B** ) Biopsy of the pancreatic tail contains a monotonous population of cells with abundant eosinophilic cytoplasm arranged in a pseudoalveolar and papillary pattern with intervening vasculature. ( **C** ) Immunohistochemistry demonstrating positive nuclear β-catenin expression in all tumor cells. ( **D** ) Macroscopic examination of the Whipple resection specimen showing a well-circumscribed tan-gray homogeneous nodule in the pancreatic head and ( **E** ) section of the distal pancreatectomy revealing a mass in the pancreatic tail extending into the splenic hilum. The mass has a tan-pink, homogeneous soft cut surface. A few matted tan-white lymph nodes ( *arrow* ) firmly adherent to the mass. ( **F** ) Histology of the pancreatic head mass featuring low-grade bland tumor cells detached from blood vessels forming fibrovascular stalks, similar to the biopsy specimen as in ( **B** ), which is diagnostic of SPN.

A 10-year-old female with a past medical history significant for multicystic dysplastic left kidney, persistent urogenital sinus, recurrent urinary tract infections, and hip dysplasia presented to the emergency department with left upper quadrant, left chest, and left shoulder pain for approximately the previous month. This patient had a height and weight of 154 cm and 37 kg at presentation, respectively. Abdominal ultrasound demonstrated a heterogenous mass at the pancreatic tail with several lesions adjacent to the spleen, raising initial concern for lymphoma or other neoplastic process. Subsequent magnetic resonance imaging (MRI) was notable for a 4.7 × 4.0 × 3.4 cm bilobed mass in the pancreatic tail and a 1.2 × 1.0 × 1.3 cm lesion in the pancreatic head. All tumor markers, including beta human chorionic gonadotropin (β-HCG), serum alpha fetoprotein (AFP), carcinoembryonic antigen (CEA), and carbohydrate antigen 19-9 (CA 19-9), were within normal limits. Biopsies were performed to ensure that both lesions were of the same origin. The pancreatic tail lesion was biopsied via a percutaneous core needle and the pancreatic head mass was biopsied via endoscopic ultrasound (EUS) with a fine needle aspiration (FNA). Histology demonstrated monotonous tumor cells admixed with capillary-sized blood vessels. Immunostains showed tumor cells positive for β-catenin, supporting the diagnosis of SPN ( Fig. 1A, B ).

The patient subsequently underwent distal pancreatectomy with splenectomy and pancreaticoduodenectomy, otherwise known as MPP. The procedure began with a midline incision. We mobilized the stomach, spleen, and pancreatic tail to identify the large palpable pancreatic tail tumor. The splenic artery and vein were ligated 1 cm proximal to the tumor margin and the pancreas was transected with an Endo GIA stapler (Endo GIA Ultra Universal Stapler, Medtronic, Minneapolis, MN) with bovine pericardium buttressing (Peri-Strips Dry, Baxter, Deerfield, IL). The spleen was unsalvageable due to extension of tumor into the splenic hilum. A standard pancreaticoduodenectomy was performed in the usual fashion without complication. During the dissection, one enlarged vena cava lymph node was excised and sent for a frozen section and found to be negative for tumor. The remaining 6 cm of the middle pancreas appeared viable and well perfused. The operation lasted 6 hours and 46 minutes and was without intraoperative complication.

On macroscopic examination, the tail mass measured 4.8 × 4.0 × 3.4 cm abutting the spleen with grossly positive lymph nodes ( Fig. 1C ) and the head mass measured 1.3 × 1.2 × 1.2 cm with negative margins. Histologically both masses shared similar morphology characteristic of solid pseudopapillary neoplasia ( Fig. 1D, E ). Five out of 34 lymph nodes were positive. Pathologic staging was mpT3N2.

The patient recovered well from surgery with a low-volume amylase-rich fluid in the surgically placed drain in the immediate post-op period, which did not require any intervention (Clavien–Dindo class I). She was discharged from the hospital on postoperative day 13 tolerating a regular diet. She continued to recover well with no evidence of endocrine or exocrine pancreatic insufficiency or recurrence for 12 months post-op. Follow-up has included clinic visits with medical oncology and a computed tomography (CT) of the chest and MRI of the abdomen/pelvis every 3 months for surveillance imaging. There has been no evidence of disease recurrence or progression to date.

## Discussion

Multicentric SPNs are very rare. To our knowledge, this is one of the first reported cases of multicentric SPN in a pediatric patient. At our institution, there have been three cases of SPN treated surgically with good results over the past 5 years ( Table 1 ). Two patients with unifocal SPN lesions were treated with pancreaticoduodenectomy, of which one patient subsequently developed both exocrine and endocrine pancreatic insufficiency. All patients have been recurrence free on follow-up.

**Table 1:** Single center experience with pediatric solid pseudopapillary neoplasm

Age (y)	Sex a	Tumor location in pancreas	Tumor size	Pathology	Surgery	Stage	Days to discharge	Complications	Endocrine function preserved?	Exocrine function preserved?	Disease-free months
14	F	Head, neck, and body	8 × 7.5 × 4.5 cm	SPN, R0 resection, 0/7 nodes positive	Pylorus-preserving pancreaticoduodenectomy	pT3N0	7	CDII: exocrine pancreatic insufficiency, type 1 diabetes	No	No	12
13	F	Head and neck	12.5 × 9.2 × 8.9 cm	SPN, R0, 0/21 nodes positive	Pancreaticoduodenectomy	pT3N0	8	none	Yes	Yes	48
10	F	Head and tail	Head: 1.3 × 1.2 × 1.2 cm Tail: 4.8 × 4 × 3.4 cm	SPN, tumor <1 mm from the margin. 5/34 nodes positive	Distal pancreatectomy and splenectomy, pancreaticoduodenectomy	pT3N2	13	CDI: low-volume pancreatic leak	Yes	Yes	12

Abbreviation: SPN, solid pseudopapillary neoplasm.

a Sex assigned at birth.

The decision to proceed with MPP was conducted with a multidisciplinary team, including general pediatric surgery, adult surgical oncology, and both pediatric and adult medical oncology. The only other previously reported case of multicentric SPN in a pediatric patient was treated with enucleation, which, by definition, does not provide an R0 resection or complete analysis of regional lymph nodes. 8 However, the evidence of a need for R0 resection is currently limited. One study showed that age less than 13.5 years and positive surgical margins predicted recurrence. 10 Other studies have suggested that R1/limited resection has similar clinical outcomes to R0 resection in these patients. 11 12 MPP provided the advantages of R0 resection, removal of involved regional lymph nodes, and preservation of pancreatic exocrine and endocrine function. Gupta et al showed in their review and case presentation that MPP had a 32.1% morbidity, 25% chance of endocrine insufficiency, and 17.8% chance of exocrine insufficiency. 13 Previously, total pancreatectomy was the surgical standard for multicentric pancreatic lesions. Total pancreatectomy offers a shorter surgical time with fewer complications than MPP, but causes insulin-dependent diabetes and pancreatic exocrine insufficiency for a lifetime. Insulin-dependent diabetes, in particular, has a significant morbidity and mortality that must be balanced with the longer, more complicated surgery of an MPP, particularly with the extended life expectancy of a child. 9

In our patient, 5 of 34 resected lymph nodes were positive on final pathology, which may support the decision for surgical resection. Due to a paucity of cases, there is no definitive predictor of metastasis or recurrent disease. One study estimates that 43.1% of pediatric patients have metastatic disease, with predictors of recurrence or metastasis including high Ki-67 proliferative index, large tumor size, and lymphovascular invasion. 11 In a 2018 review, multiple studies found lymph node positivity as a negative prognostic factor. However, other studies found no significant difference in prognosis for patients with node-positive disease. 11 13 14 15 Rare cases of extremely aggressive SPN have been reported with high-grade histologic features, including pleomorphic nuclei, extensive tumor necrosis, very high mitotic rate, a diffuse growth pattern, and one case in which the tumor contained a component of sarcomatoid carcinoma. 16 None of these features were present in the current case. Ultimately, our patient's prognosis is excellent with a greater than 95% cure rate after surgical resection and expected 97% disease-specific survival at 5 years. 1 7

Of interest, our patient has multiple congenital abnormalities, including a multicystic dysplastic left kidney and persistent urogenital sinus, which raise concern for a genetic syndrome underlying her rare presentation with multicentric SPN. Genetic testing was conducted on our patient and no known genetic syndromes or germline mutations of significance were identified. There are reported cases of SPN in patients with familial adenomatous polyposis (FAP). However, no genetic syndromes or risk factors have been specifically associated with SPN. 1 Interestingly, there are three case reports of SPN in patients with urogenital abnormalities: Bhattarai et al 18 reported a case of SPN in a child with left renal agenesis and bicornuate uterus, Guan et al 19 similarly reported SPN in a young female with solitary kidney and uterus didelphys, and Sharma et al 20 reported SPN in a child with left duplex kidney and vaginal septum. *Wnt* signaling is hypothesized to be the connection between SPN and urogenital abnormalities because the loss of *Wnt* signaling is related to *CTNNB1* mutations found in SPN and *Wnt5a* mutations have been linked to urogenital abnormalities. 17 However, given the rarity of these cases and the uncertain origins of SPN, it is difficult to draw a definitive conclusion about the connection between our patient's urogenital abnormalities and SPN.

## Conclusion

SPN is a rare low-grade malignant tumor of the pancreas, with infrequent occurrence of multicentricity. It can be found in the pediatric population, and there is increasing frequency in teenage females. We present a case of multicentric SPN treated with middle-sparing pancreatectomy in a pediatric patient. We achieved R0 resection of the primary tumors and identified positive nodal involvement. This approach should be considered when encountering future patients with multifocal pancreatic SPN disease.

## Notes

Importance for a Pediatric Surgeon This is one of the first reports of multicentric solid pseudopapillary neoplasm in a pediatric patient. Middle-preserving pancreatectomy is a viable surgical approach to patients with multiple pancreatic lesions including multicentric solid pseudopapillary neoplasm.
